# Characterisation of Host Growth after Infection with a Broad-Range Freshwater Cyanopodophage

**DOI:** 10.1371/journal.pone.0087339

**Published:** 2014-01-29

**Authors:** Siobhan C. Watkins, James R. Smith, Paul K. Hayes, Joy E. M. Watts

**Affiliations:** 1 School of Biological Sciences, University of Portsmouth, Portsmouth, United Kingdom; 2 School of Pharmacy and Biomedical Sciences, University of Portsmouth, Portsmouth, United Kingdom; University of Strathclyde, United Kingdom

## Abstract

Freshwater cyanophages are poorly characterised in comparison to their marine counterparts, however, the level of genetic diversity that exists in freshwater cyanophage communities is likely to exceed that found in marine environments, due to the habitat heterogeneity within freshwater systems. Many cyanophages are specialists, infecting a single host species or strain; however, some are less fastidious and infect a number of different host genotypes within the same species or even hosts from different genera. Few instances of host growth characterisation after infection by broad host-range phages have been described. Here we provide an initial characterisation of interactions between a cyanophage isolated from a freshwater fishing lake in the south of England and its hosts. Designated ΦMHI42, the phage is able to infect isolates from two genera of freshwater cyanobacteria, *Planktothrix* and *Microcystis*. Transmission Electron Microscopy and Atomic Force Microscopy indicate that ΦMHI42 is a member of the *Podoviridae*, albeit with a larger than expected capsid. The kinetics of host growth after infection with ΦMHI42 differed across host genera, species and strains in a way that was not related to the growth rate of the uninfected host. To our knowledge, this is the first characterisation of the growth of cyanobacteria in the presence of a broad host-range freshwater cyanophage.

## Introduction

Studies of viruses in the environment, particularly those infectious to prokaryotes, have led to important recent discoveries, including the existence of virophages [1] and viruses infectious to archaeal hosts [2], however, we still have a limited understanding of the relationships between viral lineages, or the interactions between viruses and their hosts within the environment [3].

Arguably, the most comprehensively studied group of non-medically or agriculturally important viruses, are the marine cyanophages [4]. Cyanophages infect cyanobacteria, which are globally important microorganisms found in many habitat types [5]–[7]. Cyanobacteria are major ecosystem engineers and are thought to have been responsible for the initial production of free oxygen in the Earth’s atmosphere [8]. Present day cyanobacteria are particularly important in aquatic environments, where they can be the dominant primary producers, with some taxa able to reduce nitrogen from the atmosphere and incorporate it into organic matter [9]. Cyanophages are known to influence cyanobacterial community composition, population diversity and functional activity in the oceans by contributing to host-specific mortality, thereby perturbing both primary production and biogeochemical cycling. In addition to their role in host mortality, phages can act as transducing agents, transferring genetic material between bacterial lineages, and so influencing bacterial genotypic and thus phenotypic diversity [10]. It is becoming increasingly evident, therefore, that viruses in the environment have a significant impact on microbial ecology and the role of marine cyanophages as agents of influence in marine cyanobacterial systems is important.

Cyanophages in freshwater systems have received less attention than their marine counterparts. Some freshwater and marine cyanophages may share common ancestry [11]–[12], however, cyanophages that are distinct from marine equivalents have also been described from freshwater environments [13]. The members of a number of freshwater cyanobacterial taxa produce toxins and form surface blooms in eutrophic waters that can have disastrous implications for aquatic life and water quality [14]. The control of such harmful cyanobacterial blooms (HCBs) through the application of lytic cyanophages has been considered as a potential management strategy, but deploying cyanophages as a management tool poses a number of practical problems [15], including the intensity of water treatment necessary following a sudden release of previously cytosolic toxins into the water column, the practical aspects of generating and applying large quantities of virions to a pond, lake or river, and the public perception of this type of treatment strategy.

Other issues associated with the use of cyanophages to mediate HCBs relate to the intricacies of interactions between freshwater cyanobacteria and their phages in the environment. Bacteriophages have a range of strategies for interacting with their bacterial hosts, including the capacity to infect more than one strain or species, and the nature of that infection i.e. lytic or non-lytic. Previous work [15] has highlighted the prevalence of both narrow and broad host-range cyanophages obtained from freshwater samples: having either a narrow or a broad host-range can confer advantages depending on the environmental situation. For example, in low nutrient, sparsely populated waters, where phages may not come into contact with many potential hosts, a broad-host range is likely to be advantageous, whereas in areas where host abundance is high (such as in eutrophic freshwaters) it may be advantageous to infect a narrower range of hosts, but at high efficiency. The determination of a phage’s host range is not possible without culture-based studies. This work presented here begins to extend our understanding of broad-host range cyanophages by examining the growth of diverse infected hosts under controlled conditions.

The determination of host range specificity is a well-established element of marine cyanophage characterisation [23]–[24], however, such studies tend to restrict characterisation to within, and between, *Prochlorococcus* and *Synechococcus* [25]–[26]. The ability of phages to infect a range of hosts is determined by a number of factors, including the presence of host restriction modification systems, phage genome uptake blocks and CRISPR/cas systems in the host, and attachment and adsorption mechanisms [27]–[30]. Cyanobacteria are an ancient lineage and their relationship with cyanophages is likely to have developed over the course of this long evolutionary history. This prolonged exposure of cyanobacteria to attack by cyanophages is likely to be recorded within cyanobacterial genomes [31], and will have contributed to the acquisition of robust defence and infection systems which may be collectively observed through direct examination of growth.

Host range is also important when considering a more fundamental problem surrounding the concept of phage-mediated control of HCBs, which is the genomic diversity of the bloom-forming cyanobacteria. The usual approach involves the isolation of clonal phages that can be applied directly to eutrophic waters to destroy HCBs. However, the genomic plasticity of target host cyanobacteria renders the possibility of identifying a single cyanophage, or a few cyanophages, that are lytic to all genotypes within a bloom very low. For example, *Microcystis* and *Planktothrix* communities have both been shown to encompass a variety of ecotypes that differ genetically and phenotypically, including in characteristics such as toxicity, presumably dependent on the ecological niche that they occupy [16]–[19]. Therefore, a single cyanophage is unlikely to be able to act as a lytic agent against a genetically diverse and constantly changing cyanobacterial population i.e. a cyanophage infectious or lytic against one ecotype of *Microcystis*, may not be infectious to another [20]. Furthermore, rates of co-antagonistic evolution observed between phages and bacteria will likely influence defence and attack strategies very quickly. The presence of temperate viruses, lysogens and pseudo-lysogeny in the environment [21]–[22], add a further level of complexity to the study of the ecology of freshwater viruses and their hosts. The standard methods for analysis of phage growth kinetics rely heavily on plaque assay based methods such as the one-step growth curve [32]. While plaque assays are possible for some planktonic cyanobacteria, such as members of the genera *Anabaena* and *Microcystis*, members of the ecologically important genus *Planktothrix* display gliding motility, which precludes plaque formation on solid media. Initial genetic characterisation of cyanophages has been performed previously using primers designed for the amplification of structural genes similar to those found in the myovirus coliphage T4. Myoviruses, however, are just one component of the virioplankton and, moreover, they are known to be widely divergent [33], and so current PCR-based molecular analyses impose significant constraints on the characterisation of newly isolated freshwater cyanophages. Therefore, the standard method for genetic characterisation of new phages is, increasingly, *de novo* whole genome sequencing (WGS) [34], [35], [36]. While WGS is a powerful technique, inaccessibility due to cost and the inherent difficulties associated with genome assembly and analysis, can be issues. These collective limitations, i.e. the inability to rely on plaque assays and lack of primers for rapid initial genetic characterisation, are, in part, responsible for the paucity of information about freshwater cyanophages.

Classification of phage morphology is typically assessed using transmission electron microscopy (TEM), however, preparation and visualisation of phages can be difficult [37]. Atomic force microscopy (AFM) is slowly emerging as an alternative to TEM for the determination of viral morphology, however to date it has only been used to image marine cyanophages [38]. AFM provides the potential to convert highly resolved digital images to quantitative data [39].

In this study we provide an initial characterisation of host growth after infection of cyanobacterial strains from multiple genera with ΦMHI42, a freshwater cyanophage originally isolated on *Microcystis aeruginosa* BC84/1. Using a combination of both TEM and AFM, we show that ΦMHI42 is a member of the Podoviridae. We demonstrate that this cyanophage has a broad host-range, in that it is able to infect both *Planktothrix* and *Microcystis*, and that the impact of infection on the growth of the host is variable between strains and across genera. To the best of our knowledge, this is the first description of the growth characteristics of cyanobacterial host cultures following infection with a broad host range freshwater cyanophage.

## Materials and Methods

### Host Strains and Growth Conditions

All cyanobacterial cultures used in this study were non-axenic. *Microcystis aeruginosa* BC84/1 (University of Bristol, UK), was grown in the open laboratory in BG11 medium [40] at room temperature (∼20°C) and away from direct sunlight, at a constant incident irradiance of between 10–15 µmoles m^−2^ s^−1^. Additional cyanobacterial strains used for host range assays were grown at constant temperature under cool white fluorescent light in incubators as follows: *Microcystis aeruginosa* 1450/8 (CCAP, Argyll, Scotland), was grown in BG11 medium at 20°C at an incident irradiance of 8–10 µmoles m^−2^ s^−1^; *Planktothrix rubescens* 9316 and *Planktothrix agardhii* 137 (University of Bristol, UK), which were grown in Oscillatoria medium [41] at 20°C at incident irradiance of 2–3 µmoles m^−2^ s^−1^ for *P. rubescens* and 6–7 µmoles m^−2^ s^−1^ for *P.agardhii*; *Anabaena flos-aquae* (University of Bristol, UK) was grown in Oscillatoria medium at 20°C at an incident irradiance of 10–15 µmoles m^−2^ s^−1^.

### Initial Cyanophage Isolation

A surface water sample (5 L) was collected from a fishing lake in Hayling Island, Hampshire, UK (SZ 73658 98822) on 19th September 2011. No official permission was required to sample as the lake is a public water body. Aliquots of *M. aeruginosa* BC84/1, in early exponential growth (1.5 ml), were mixed with 200 µl of the water sample and left for 24 hours, under standard host growth conditions. The host/sample mixtures were then mixed with 1.5 ml sterile low-melting temperature agarose (0.8% w/v) at 37°C, and poured onto the surface of a BG11 agar (0.8% w/v) plates, incubated (as above) and checked for the formation of plaques. Plaques (in agar plugs) were removed from the plate and stored in cyanophage (CP) buffer (5 mM MgCl_2_, 5 mM CaCl_2_, 10 mM NaCl, 10 mM HEPES, pH 7), at room temperature in the dark, before being subjected to three rounds of purification (plating followed by plaque isolation, as described above).

### Host Range Determination

Samples of phage-induced cyanobacterial lysate (200 µl) were combined with exponential phase cyanobacterial cultures (1.5 ml) in a multiwell tissue culture plates (well volume 3 ml). Additional growth medium was added (1 ml) and the plates were incubated as above and checked daily for the presence of cleared lysates. The lysate from cleared wells was removed and centrifuged at 20 000×g for two hours. Subsequently, the supernatants were collected and stored at room temperature in the dark. Where possible, cyanobacterial lawns (*M.aeruginosa*) were infected with ΦMHI42 and plaque assays which had been left to progress to the extent that no cyanobacterial host remained visible on the plate were kept for further examination. The resulting overgrowth of bacteria, associated with non-axenic growth, was examined for further plaque formation, and therefore the potential for infective capacity on heterotrophs.

### Transmission Electron Microscopy (TEM)

Purified viral lysate was applied to pioloform coated copper grids and left to dry at room temperature. Samples were positively stained with 2% (w/v) uranyl acetate [42] and observed at 80 kV using a Jeol JEM 2100 Transmission Electron Microscope.

### Atomic Force Microscopy

Poly-L-lysine (5 µL, 0.01% (w/v), mol wt 7000–150000) was placed on freshly cleaved muscovite mica (Agar Scientific, Stansted, Essex, UK), left for 2 minutes and the mica was then dried in a stream of N_2_ at room temperature. Viral lysates were diluted (1∶10) in ultrapure water (18 MΩ; Sigma-Aldrich) and placed (5 µL) on the mica surface, left for 2 minutes after which glutaraldehyde (5 µL; 4% (w/v) in 0.2 M sodium cacodylate) was added. After a further 2 minutes, the surface was extensively rinsed with ultrapure water and dried as above. The mica was then mounted on a nickel disc (1 cm diameter) with double-sided adhesive tape and placed on the AFM scanner. AFM studies were performed using a Multi-Mode/NanoScope IV Scanning Probe Microscope (Bruker, Santa Barbara, CA, USA) in air under ambient conditions (T = 24°C, RH = 45%) using the J-scanner (max. xy = 200 µm). Scanning was performed in Tapping Mode using a Si cantilever with integrated tip (t_nom_ = 3.6–5.6 µm, l_nom_ = 140–180 µm, w_nom_ = 48–52 µm, υ_nom = _200–400 kHz, υ_meas_ = 333.7 kHz, k_nom_ = 12–103 Nm^−1^, R_nom_<10 nm; Model: OTESP, Bruker, France) and an RMS amplitude of 2.0 V. Images were subsequently processed (plane fitted and shaded using an artificial light source) using NanoScope analysis software (V 1.40, Bruker, CA, USA).

### PCR Amplification

Viral-induced host cell lysates were used as a template for amplification reactions performed using published protocols and primer sets previously employed to characterise isolated and environmental samples of marine cyanophage (Table 1).

**Table 1:** Primer sets used in this study. Primer sequence and PCR conditions are provided in the references.

Primer set designation	Target	Reference
CAP	g23 gene(Major Capsid Protein)	[43]
CPS	g20 gene(Coliphage Portal Vertex Protein)	[44]
CPS8.1/1.1	g20 gene(Coliphage Portal Vertex Protein)	[45]
DNAPol	Podovirus DNAPolymerase	[46]

### Growth of Hosts in the Presence of Cyanophage

The growth of cyanobacterial hosts was assessed using a turbidometric method; these data were used to compare growth characteristics of each culture after infection with ΦMHI42 [47]. For each host strain, nine replicate cultures were inoculated with ΦMHI42 at a multiplicity of infection (m.o.i.) of 5 or 0.5: phage titre was determined using *M. aeruginosa* BC84/1 as host and performed according to standard method based on viral quantification by plaque forming units [32]. Absorbance of the cultures was measured at 660 nm and 750 nm at 24 hour intervals throughout the infection cycle: cultures were infected with ΦMHI42 at the start of exponential growth. Uninfected cultures were used as controls.

## Results

### Characterisation of ΦMHI42

**Figure 1:**
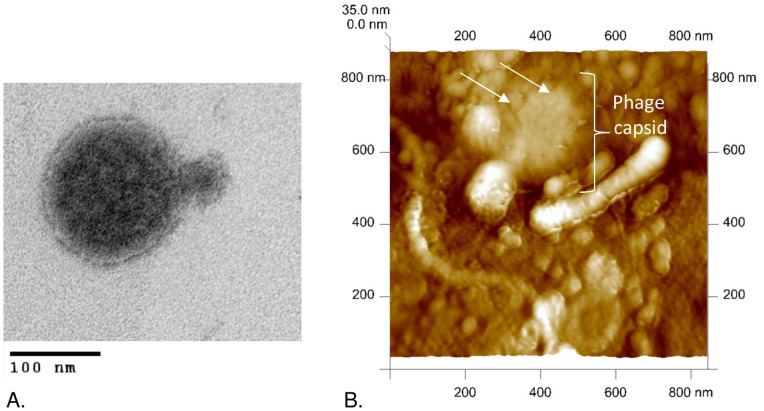
φMHI42 viewed at high magnification using: A. TEM and B. AFM. A shows a single phage, similar in morphology to the family *Podoviridae*. B shows a single phage among associated cell debris, the presence of a viral capsid confirms size and morphology of the structures observed in A. Repeating units on the capsid are highlighted with arrows.

TEM analysis revealed a cyanophage displaying characteristics most similar to those of the family *Podoviridae* (Fig. 1A). ΦMHI42 appears to possess a short, non-contractile tail, however, the capsid, with a diameter of approximately 100–120 nm, is considerably larger than for most previously described podoviruses (usually 40–60 nm). Capsid size was confirmed using AFM (Fig. 1B): lighter areas in the AFM image represent areas raised above the substrate surface. The image shows repeating, discrete units on the surface of the capsid, which had an average diameter of 34 nm (340 Å), as determined with the Nanoscope analysis package.

It was not possible to generate PCR amplicons from φMHI42-induced cell lysates using marine cyanophage-specific primer sets (Table 1) which is consistent with previous studies [15]. The highly degenerate primer set CPS 8.1/1.1 (45) primed the amplification of a fragment of about the expected size, but this amplicons lacked any sequence similarity to any previously characterised bacteriophage derived nucleotide or protein sequences (data not shown).

### Host Growth Characteristics after Infection with ΦMHI42

**Figure 2:**
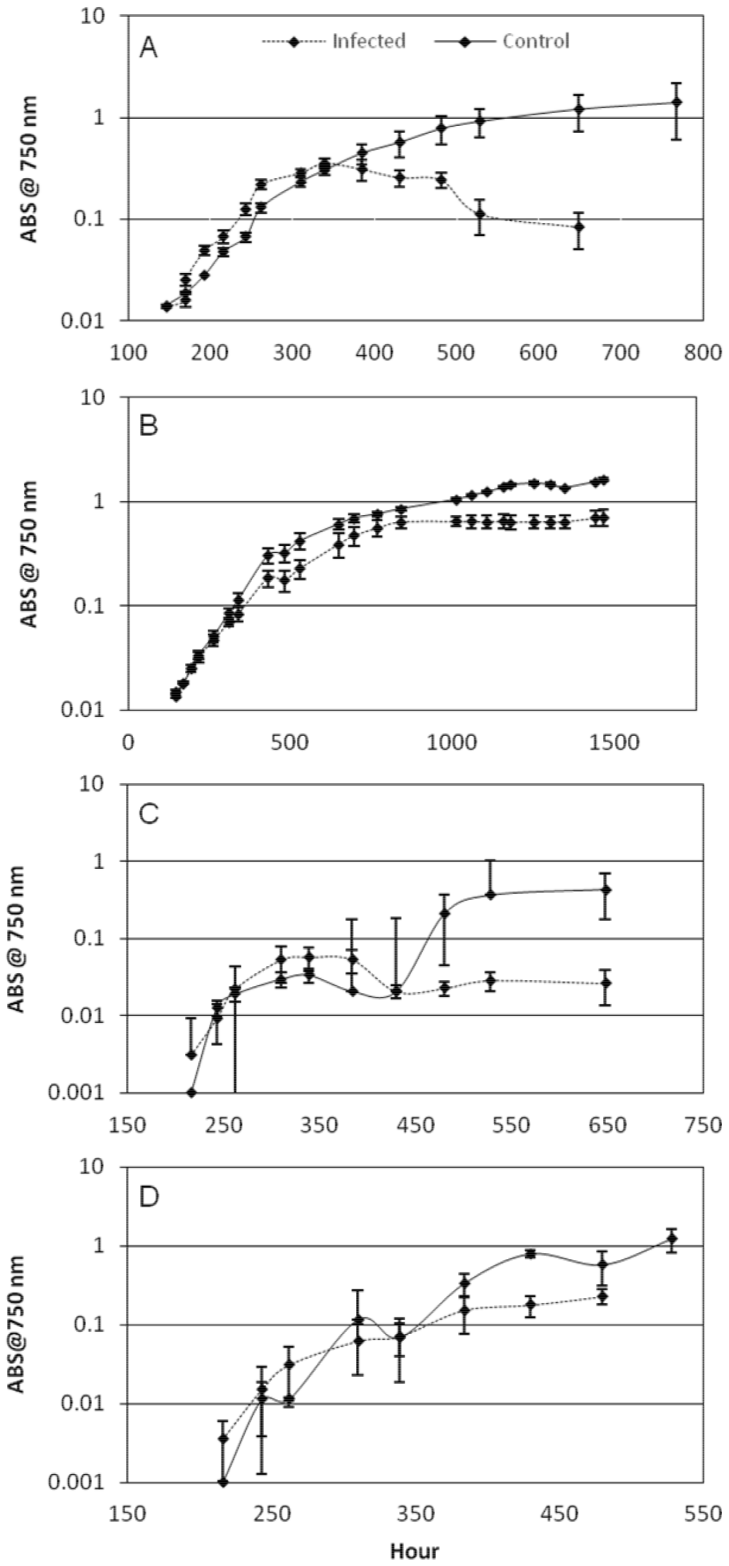
Lysis profiles generated by infecting five strains of cyanobacteria with φMHI42, as determined by measuring absorbance at 750 nm – *M. aeruginosa* BC84/1 (A), *M. aeruginosa* 1450/8 (B), *Planktothrix agardhii* 137 (C), *Planktothrix rubescens* 9316 (D). Profiles begin at point of infection and are plotted on a logarithmic scale. Dashed lines are infected culture series, error bars represent standard deviation over three replicate cultures. The solid line represents triplicate control cultures, which were inoculated with the host but not subsequently infected with phage.

In host range specificity tests, ΦMHI42 was used to infect cultures of *Microcystis*, *Planktothrix* and *Anabaena*, and also tested for infective capacity against heterotrophic bacteria associated with the non-axenic cultures. Cultures of both *Microcystis* and *Planktothrix* were susceptible to lytic infection, but associated bacteria and cultures of *Anabaena* were not. Host growth profiles (Fig. 2) demonstrate the progression of infection in replicate cultures established from the same inoculum and using a m.o.i. of 5. Growth of the hosts in the uninfected controls progressed as expected, where the large error bars are associated with the variability in growth commonly seen in cultures where the cells (*Microcystis*) or filaments (*Planktothrix*) form clumps.

**Figure 3:**
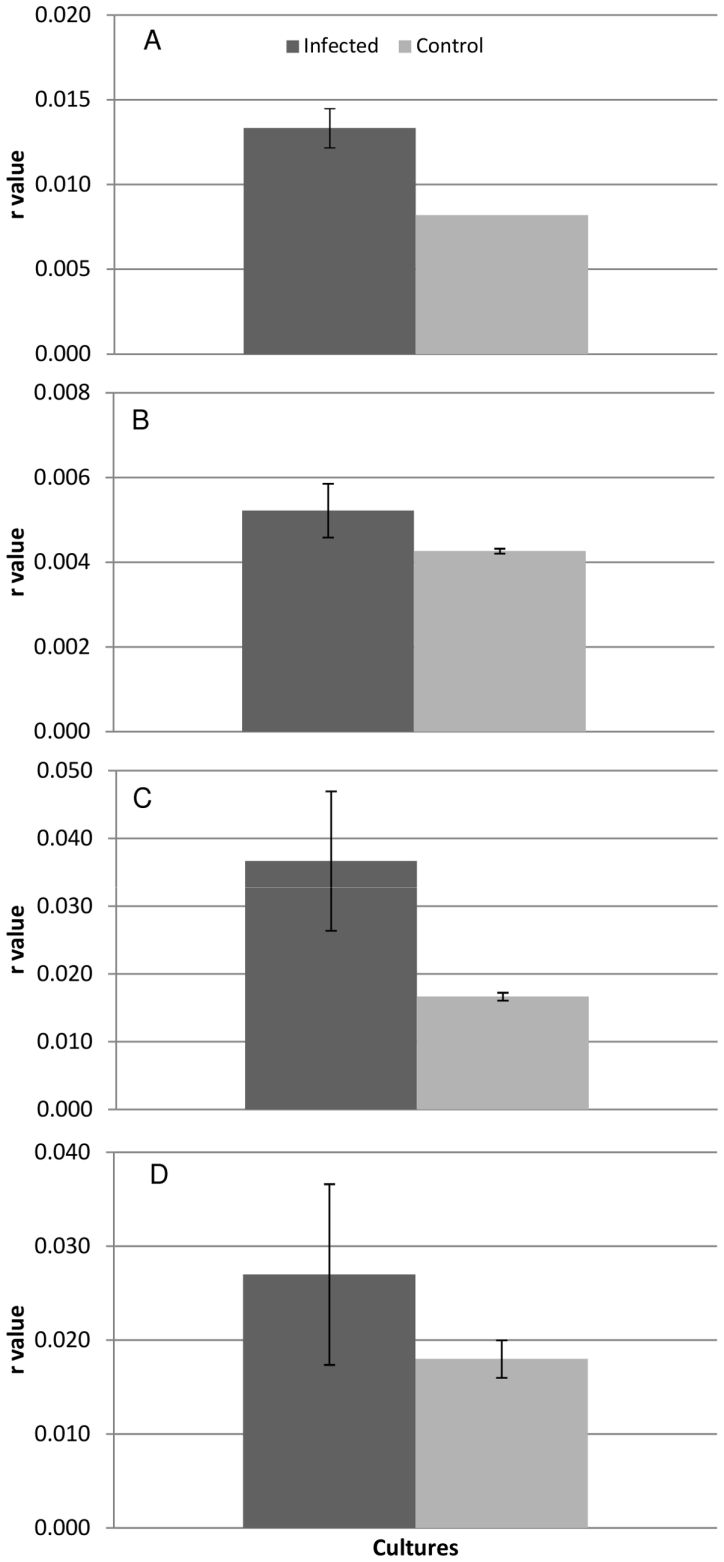
Statistical analysis based on mean r values for replicates cultures of each host strain infected by ΦMHI42 - *M. aeruginosa* BC84/1 (A), *M. aeruginosa* 1450/8 (B), *Planktothrix agardhii* 137 (C), *Planktothrix rubescens* 9316 (D). Error bars represent standard deviation. Growth rates were assessed for significant difference with the ANOVA test and differences separated with a Bonferroni corrected t-test.

The growth profile of *M. aeruginosa* BC84/1 infected with ΦMHI42 (Fig. 2A) shows a clear onset of lysis after about 400 hours and subsequent drop in cell concentration (absorbance) across all replicates, yielding a profile similar to that observed for fast-growing lytic phage-infected bacteria, for which this technique has been previously used. This profile results from the step-wise release of phage from the host cell [48]. Although it is difficult to determine a latent period from the growth curve generated, it is possible to compare growth characteristics between different host strains infected with ΦMHI42. Infection of BC84/1 with ΦMHI42 resulted in a halt in exponential growth of the host after approximately ten days. In contrast to BC84/1, infection of *M. aeruginosa* 1450/8 with ΦMHI42 resulted in retardation of host growth as opposed to catastrophic lysis: the infected *M. aeruginosa* 1450/8 continued to grow, but at a reduced rate and achieving a lower cell concentration in stationary phase in comparison to the control cultures (Fig. 2B). At this time, the cultures did not appear to have become scenescent, however, samples removed from the cultures also subsequently produced lysis in a culture of BC84/1. The growth profile on this host was suggestive of a chronic infection strategy. Assessment of exponential growth rates (r) also showed differences in infection profile between the two strains of *M. aeruginosa* (Fig. 3A & B). Analysis of r values of all replicates showed that BC84/1 cultures infected with φI42 had significantly higher exponential growth rates than the uninfected controls. This can also be seen on the lysis profile (Fig. 2A). *M. aeruginosa* 1450/8 replicate cultures also demonstrated a significant, but less pronounced, difference in exponential growth rate between infected and control cultures.

Laboratory cultures of *Planktothrix* species often demonstrate variable growth patterns, such as a tendency to clump, in ways that seem to be unrelated to changes in standard growth conditions. For this reason the growth curves of the uninfected cultures (Fig. 2C & D) were less reproducible than for *Microcystis*. Infection by ΦMHI42 resulted in almost immediate effect on the growth of the host strains (Fig. 2C & 2D). The onset of lysis was rapid and highly reproducible across all replicates. Cultures of *P. agardhii* 137 required approximately five days after infection to stop growing completely (Fig. 2C), considerably shorter than for *Microcystis.* Statistical analyses of exponential growth rates of replicate infected and triplicate control cultures were difficult to interpret due, again, to variability in host growth. Assessment of *P. agardhii* 137 showed a significant increase in growth rates of the infected group compared to the control group (Fig. 3C), with evidence for increased variability in growth rate between infected replicates versus the control. The growth profile generated for *P. rubescens* 9316 (Fig. 2D) is misleading: due to the rapid growth of heterotrophic bacteria in response to lysis of *P. rubescens*, absorbance begins to increase after hour 400, despite complete lysis of the red-pigmented cyanobacterium. No significant difference in exponential growth rates between infected and uninfected cultures was observed (Fig. 3D), possibly because of the overgrowth by other bacteria.

### Effect of Lowering Multiplicity of Infection

To assess the effect of m.o.i on lysis profiles, growth of *Planktothrix* was also assessed at a nominal m.o.i. of 0.5. At this reduced m.o.i. there was no impact on the growth of the host (data not shown), which is consistent with the results from previous studies [49].

## Discussion

The genetic richness and diversity in freshwater bacteriophage communities may equal or even surpass, that found in marine environments [13], [50]–[51]. Bacteriophages are known to act as important agents of mortality, as genetic reservoirs and transfer agents within bacterial communities, the impact of which will depend on their ability to infect a range of species and host ecotype, and also on whether they adopt lytic or lysogenic modes of infection. A number of effective deep sequencing techniques have been developed for the characterisation of viral communities in the environment, however, it is only by isolation and laboratory propagation that host range can be determined and some lifestyle characteristics, such as the ability to lysogenise the host, assessed.

The infection characteristics of phages and their hosts can be determined using a number of standard techniques, the majority of which are based on the development of viral plaques on lawns of host cells [52]. While such techniques generally work well with bacteria that grow reliably on solid media and in axenic culture, the characterisation of phages of hosts that cannot be grown on plates or in the absence of other microorganisms presents challenges over and above those normally experienced when working with fastidious and/or slow growing bacteria. Although generally amenable to growth in laboratory culture, cyanobacteria have relatively long generation times and some, such as *Planktothrix*, clump in liquid culture and exhibit gliding motility. The isolation of freshwater cyanophages on *Planktothrix* and other hosts does not, however, appear to be problematic, but, sustained propagation and maintenance of viability of freshwater cyanophages is challenging, particularly in relation to maintaining phage viability [53]–[54]. The issues around sustained viability are not uncommon, but they add a further level of complexity when attempting to characterise phage [55].

These challenges are reflected in the lysis profiles generated from infecting four strains of cyanobacteria with ΦMHI42. Results that are typical of lytic infection were obtained using the original host, *M. aeruginosa* BC84/1, a unicellular cyanobacterium that has been in culture since 1984. BC84/1 was the only strain in this study on which plaques form reliably, and so was used to generate the viral inocula used for all infections. The time taken for cultures to die, estimated from the growth curve, reflects the inherent slow growth rate of the host (r = 0.0082±0.0 hr^−1^). Of interest is the effect that exposure to ΦMHI42 appears to have on the growth rate of the host. For *M. aeruginosa* BC84/1, during the exponential period prior to lysis the average growth rate increased in comparison to the uninfected controls from 0.0082±0.0 hr^−1^ to 0.013±0.0012 hr^−1^. A similar result was observed for *M. aeruginosa* 1450/8 on a smaller, but nonetheless significant scale (from 0.0043±0.00006 hr^−1^ to 0.013±0.0049 hr^−1^), and which also demonstrated the slowest growth rates of all the host strains. It has been previously documented that phage infection can induce alterations in metabolic activity of their hosts [56], particularly in processes such as photosynthesis [57] which may ultimately be beneficial to the phage. That higher growth rates in these infected hosts relates directly to the presence of this phage requires confirmation. *M. aeruginosa* 1450/8 produced a very different growth profile in comparison to *M. aeruginosa* BC84/1 when incubated in the presence of ΦMHI42: there was no defined onset of lysis, and while there was a discernible colour change in the infected cultures (from blue-green to pale yellow), suggesting the establishment of lysis, a clear lysate was not produced (the lysate was, however, infectious to, and consequently produced lysis in, BC84/1). This lack of defined lysis continued in the infected cultures even through the period when the parallel controls went through stationary phase and senescence. In further comparison to BC84/1, 1450/8 grew particularly unreliably on solid medium, and was often outcompeted by heterotrophic bacteria associated with the non-axenic culture: plating appeared to strongly select for their growth as opposed to that of the cyanobacterium.

The growth of *M. aeruginosa* 1450/8 uninfected controls was more reproducible than for the other host strains, evident from the small size of the error bars, but, infection appeared to slightly increase the levels of variation between replicates. The instability observed during the infection cycle of *M. aeruginosa* 1450/8 may have resulted solely from the overgrowth by the normally background-level bacterial contaminants present in these non-axenic cultures, however, it may also relate to the unstable nature of the type of phage growth that allows the simultaneous survival of the host and the production of viable virions. Retardation of growth in *M. aeruginosa* 1450/8 in response to infection, as opposed to acute lysis, may suggest a state of chronic infection or pseudo-lysogeny [58]. The marked difference between the growth of the two strains of *Microcystis* in the presence of ΦMHI42 may relate to a number of factors associated with genetic diversity within the genus [59], however, differences in phage resistance between these two strains could also be a relic of their historical exposure to very different phage-related selection pressures imposed prior to isolation into culture [60]–[61]. ΦMHI42 was initially isolated on *M. aeruginosa* BC84/1, and was purified by continuous rounds of infection on this strain. It is possible that this purification process selected for variants of ΦMHI42 that were better adapted for growth on *M. aeruginosa* BC84/1 than the original isolate, but given that ΦMHI42 retained the ability to infect both *Planktothrix* and a further *Microcystis* this seems unlikely.

Despite having been isolated on *Microcystis* BC84/1, ΦMHI42 was more virulent against *Planktothrix*, a filamentous cyanobacterium. Previously, it has not been possible to examine a clonal phage in relation to *Planktothrix* [15], due to its capacity to glide and therefore inability to produce plaques on solid medium. In this case we were able to use the lysis profiling technique as ΦMHI42 had already been rendered clonal on its original host, *M. aeruginosa* BC84/1. While the rate of lysis is expected to be related to the normal growth rate of the host [32], [62], the time taken for the two *Planktothrix* cultures to stop growing was significantly shorter than was observed for the *Microcystis* infections. It is possible that the observed differences are related to the filamentous nature of *Planktothrix*, with rapid transmission of infection between cells along the length of the filament. If this is the case, then it would also have implications for the spread of infection in the natural environment. While assessment of host growth rate was performed for both *Planktothrix* cultures, it was difficult to draw conclusions from the data collected for *P.rubescens 9316*. For infected cultures of *P. agardhii* 137, however, infection did produce a significant increase in rate of growth compared to the control, although with more variability between replicate infected groups than was observed for *Microcystis*.

That four different strains of cyanobacteria can exhibit different responses to infection by the same cyanophage, is likely to be related to the concepts of ‘ecotypes’ and niche separation in the environment [63]. The existence of ecotypes has been clearly demonstrated in cyanobacteria, where strains with similar or identical 16S rRNA gene sequences can demonstrate considerable differences in phenotype [64]. In part, the niche occupied by particular ecotypes can be defined by the composition of phage community, i.e. resistance or susceptibility of individual cyanobacterial lineages to phage communities will determine whether or not they are able to grow at that location. The interaction between the host and any infecting phage is dynamic and often described as an “arms race” [65] where the level of exposure to the host and to other phages infecting the same host may all play a role in phage infection dynamics, which in turn will influence the evolution of host defence against phage attack. How the host response to attack by phage is likely to have developed is less clear, and further investigation is warranted to assess the presence of host restriction modification enzymes [66]–[67] or CRISPR/cas systems [68]–[69] in resistant strains.

The presence and overgrowth of contaminating bacteria in response to lysis of the dominant member of a non-axenic culture highlights further difficulties associated with examining cyanobacterial infections and subsequent physiological aspects of phage characterisation. Cyanobacteria obtained from culture collections are rarely axenic, and growth conditions selecting against the growth of the cyanobacterium will often result in rapid growth of the contaminants [70]–[71]. Non-axenic overgrowth was observed in three of the four cyanobacterial cultures used for lysis profiles, *P.agardhii* being the only culture not demonstrating this trait.

In the environment, harmful cyanobacterial blooms are likely to be affected by viral-induced host mortality, and the capacity of phages to drive genetic diversification through horizontal gene transfer will influence the generation of new cyanobacterial ecotypes and the genotypic composition of toxic blooms populations. Genetic diversification may be mediated across genera by broad host-range phages. The nature of interactions between generalist phages, specialist phages and their hosts are not well characterised, and neither are the impacts that these may have on the phage communities that could influence the formation, structure and disruption of blooms. If a phage, such as ΦMHI42, is capable of removing hosts from multiple genera, this may have impact on the general level of phage diversity. Similarly, if a phage is capable of inducing a chronic growth phase in a host present in the environment, this suggests resistance to such a phage will be generated quickly. Therefore, it is likely that the composition of both the phage and host communities is extremely dynamic, but it seems certain that interactions between the two will be important in shaping the ecology of freshwaters.
